# Monodisperse Carbon Nanospheres with Hierarchical Porous Structure as Electrode Material for Supercapacitor

**DOI:** 10.1186/s11671-017-2318-z

**Published:** 2017-09-25

**Authors:** Xiutao Yang, Hui Xia, Zhongguan Liang, Haiyan Li, Hongwen Yu

**Affiliations:** 1 School of Physics and Electronics, Central South University, Changsha, 410083 China; 2 Key Laboratory of Wetland Ecology and Environment, Northeast Institute of Geography and Agroecology, Chinese Academy of Sciences, Changchun, 130102 China

**Keywords:** Carbon nanosphere, Monodisperse, Porous materials, Energy storage and conversion, Supercapacitor, 81.05.Uw, 88.80.Fh, 82.47.Uv

## Abstract

Carbon nanospheres with distinguishable microstructure were prepared by carbonization and subsequent KOH activation of F108/resorcinol-formaldehyde composites. The dosage of triblock copolymer Pluronic F108 is crucial to the microstructure differences. With the adding of F108, the polydisperse carbon nanospheres (PCNS) with microporous structure, monodisperse carbon nanospheres (MCNS) with hierarchical porous structure, and agglomerated carbon nanospheres (ACNS) were obtained. Their microstructure and capacitance properties were carefully compared. As a result of the synergetic effect of mono-dispersion spheres and hierarchical porous structures, the MCNS sample shows improved electrochemical performance, i.e., the highest specific capacitance of 224 F g^−1^ (0.2 A g^−1^), the best rate capability (73% retention at 20 A g^−1^), and the most excellent capacitance retention of 93% over 10,000 cycles, making it to be the promising electrode material for high-performance supercapacitors.

## Background

Supercapacitors are the promising energy storing device due to their high power density, fast charge time, and long-term stability. The performance of supercapacitors greatly depends on the structure of electrode materials [1]. Due to the large surface area, unique pore structure, and good chemical and mechanical stability, carbon materials show great potential application to catalysis [2], adsorption [3], and supercapacitors [4, 5]. The nanostructured carbon materials are always designed to improve the performance of supercapacitors [6, 7].

In this case, carbon fiber [8], carbon film [9], and carbon sphere [10–16] containing porous structure are synthesized for fabricating electrode of supercapacitors. Compared to carbon spheres, carbon fiber or film suffers from lacking of three dimensioned interconnected architecture which was proved to have advantage of the charge storage and transfer. Many works have been done to produce microporous carbon spheres [10, 11], worm-like mesoporous carbon spheres [12], and ordered mesopore carbons [13–15]. Those carbon spheres with different structure all show good electrochemical performance. However, the effect of different structure is not studied systematically due to these carbon spheres with different structure prepared in diverse synthesis system.

In the paper, by using the same protocol with different dosages of triblock copolymer Pluronic F108 as template, we prepare three kinds of carbon nanospheres with distinguishable microstructure, namely monodisperse carbon nanospheres (MCNS), polydisperse carbon nanospheres (PCNS), and aggregated carbon nanospheres (ACNS). We find that the electrochemical performance varies with different carbon nanospheres. The MCNS sample shows the highest specific capacitance of 224 F g^−1^ (0.2 A g^−1^), the best rate capability (73% retention at 20 A g^−1^), and the most excellent capacitance retention of 93% over 10,000 cycles. More importantly, the synergetic effect of mono-dispersion spheres and hierarchical porous structures contribute to the better electrochemical performance of MCNS.

## Methods

### Synthesis of Carbon Nanospheres

F108/resorcinol-formaldehyde composites were synthesized by hydrothermal reaction with triblock copolymer Pluronic F108 (Mw = 14,600, PEO_132_-PPO_50_-PEO_132_) as template and phenolic resin as carbon source. Then, monodisperse carbon nanospheres (MCNS) were obtained via the carbonization of as-prepared composites, followed by KOH activation. In a typical synthesis, 0.9 g of F108 was firstly dissolved in 30 ml of deionized water forming clear solution. Then, 1.2 g of phenol and 4.2 ml of formalin aqueous solution (37 wt%) were mixed in 30 ml of NaOH solution (0.1 M) for reaction at 70 °C. After 0.5 h, the prepared F108 solution was added and the mixed solution was stirred at 66 °C for another 10 h until the deposit was observed. The obtained solution was diluted to three times and underwent hydrothermal reaction at 130 °C for 24 h. After collection and rinse, the products were carbonized at 700 °C for 3 h, denoted as intermediate carbonized carbon nanospheres for MCNS (mCNS). Subsequently, mCNS were activated with KOH in mass radio of 1:2 at 700 °C for 1 h to obtain MCNS samples. The final products of PCNS and ACNS are obtained with 0.6 and 1.8 g triblock copolymer Pluronic F108 by the same protocol. The stir time of mixed solution for PCNS and ACNS is 5.5 and 15 h, respectively.

### Microstructure Characterization

The morphology of samples was characterized by scanning electron microscopy (SEM; HELIOS Nanolab 600i) and transmission electron microscopy (TEM; Tecnai G2 F20 STWIX). The pore structure of samples was analyzed by nitrogen adsorption-desorption measurements using the accelerated surface area and porosimetry system (ASAP 2020) at 77 K.

### Electrochemical Measurement

The electrochemical performance of samples was tested by the electrochemical workstation (CHI660E). The working electrode contained MCNS, acetylene black, and poly (tetrafluoroethylene) with mass ratio of 80:10:10. Each 1-cm^2^ working electrode contained approximately 3 mg MCNS. The same fabrication method was used to prepare PCNS and ACNS electrode. The three-electrode system was constructed by as-prepared working electrode, platinum foil as counter electrode, and Hg/HgO as reference electrode in KOH aqueous solution (6 M). Cyclic voltammetry (CV), chronopotentiometry (CP), and electrochemical impedance spectroscopy (EIS) techniques were carried out to investigate the electrochemical performances of MCNS, PCNS, and ACNS.

## Results and Discussion

### Morphology

**Fig. 1:**
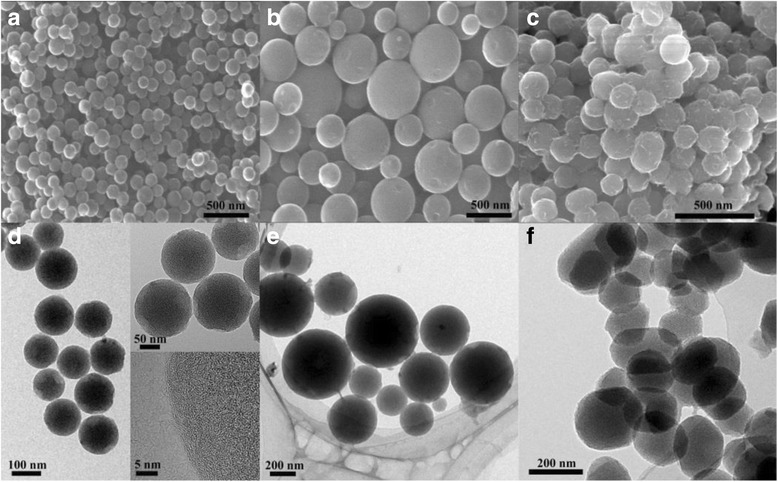
Morphology of all samples. SEM images of **a** MCNS, **b** PCNS, and **c** ACNS; TEM images of **d** MCNS at different magnification, **e** PCNS, and **f** ACNS

The morphology of samples was studied by SEM and TEM and is given in Fig. 1. From the SEM images of MCNS, PCNS, and ACNS (Fig. 1a–c), MCNS and PCNS possess well-spherical morphology but ACNS are the aggregate of irregular-shaped carbon. Moreover, the obtained MCNS are homogeneous in size (140 nm in diameter) but PCNS are in wide size distribution. The TEM images of MCNS, PCNS, and ACNS further demonstrate their microstructure. From Fig. 1d, MCNS are monodisperse carbon nanospheres and the HRTEM analysis presents the hierarchical porous structures of MCNS. As shown in Fig. 1e, PCNS are polydisperse. In addition, Fig. 1f shows the ACNS are firmly agglomerated and nondispersible. It is clear that the dosage of F108 has great impact on the size distribution and dispersibility of final products.

### Pore Structure Analysis

**Fig. 2:**
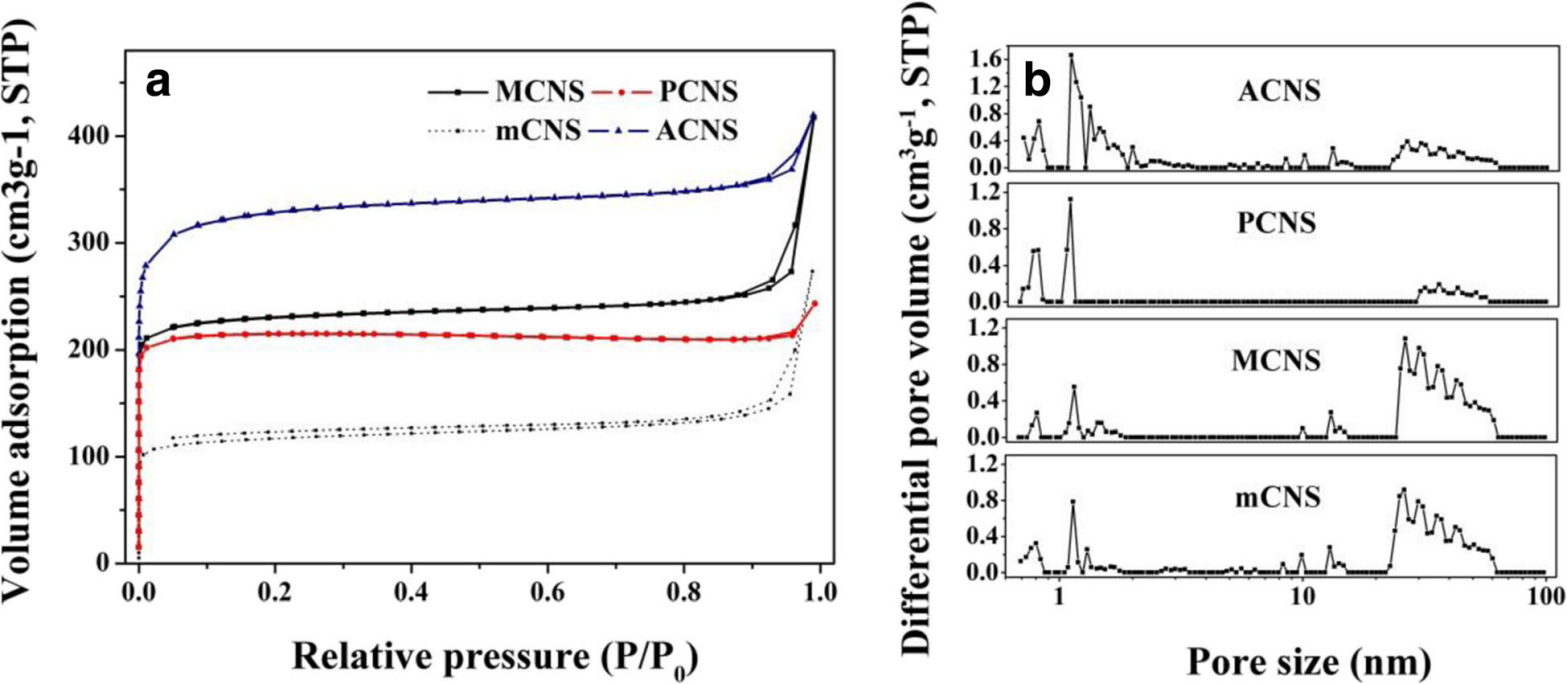
N_2_ adsorption-desorption measurements of all samples. **a** N_2_ adsorption/desorption isotherms and **b** pore size distribution

The pore structure of all samples was estimated by N_2_ adsorption-desorption measurements, summarized in Table 1. The PCNS sample shows typical microporous structure, while mCNS, MCNS, and ACNS samples present hierarchical porous structure. From Fig. 2a, all samples show the pseudo-type I isotherm with steep uptakes below *P*/*P*_0_ = 0.01, suggesting existence of plenty of micropores. The H3 hysteresis loops at high relative pressure can be observed at the isotherm of mCNS, MCNS, and ACNS, suggesting the existence of interstice-type pore structure which is mainly resulting from the voids between individual particle and the mesopores. The pore distribution curves (Fig. 2b) intuitively demonstrate the microporous structure of PCNS and also the co-existence of developed micropore and mesopore in mCNS, MCNS, and ACNS. It is interesting to notice that the mCNS sample shows similar N_2_ adsorption/desorption isotherms and pore size distribution curve to that of MCNS, indicating that their pore structure are similar. However, the pore volume of mCNS (0.423 cm^3^ g^−1^) is lower than that of MCNS (0.645 cm^3^ g^−1^). Thus, KOH activation contributes to the hierarchical porous structure of MCNS by increasing the pore volume. Compared to MCNS, the pore volume of PCNS (0.37 cm^3^ g^−1^) sharply decreases with negligible mesopore and ACNS present the similar pore volume (0.649 cm^3^ g^−1^) with decreased mesopore. The significant mesoporosity of MCNS are mainly due to the loosely agglomerated monodispersed carbon nanospheres. It is obvious that polydispersity of PCNS and aggregate of ACNS goes against the formation of mesopore between individual particles. Adding the F108 mainly causes microporous PCNS transforming to hierarchical porous MCNS by keeping the uniform size of carbon nanospheres. However, excessive F108 results in the aggregate of carbon nanospheres. Obviously, the porous structure difference of PCNS, MCNS, and ACNS is mainly caused by the adding of F108.

**Table 1:** The specific surface area and pore volume parameters

Samples	*S*_BET_^a^/m^2^ g^−1^	*S*_micro_^b^/m^2^ g^−1^	*V*_total_^c^/cm^3^ g^−1^	*V*_micro_^d^/cm^3^ g^−1^	*V*_mesol_^e^/cm^3^ g^−1^
MCNS	703	610	0.645	0.317	0.328
PCNS	646	621	0.37	0.32	0.05
mCNS	361	289	0.423	0.265	0.158
ACNS	1008	819	0.649	0.424	0.224

^a^Specific surface area calculated by BET method

^b^Specific surface area of micropores

^c^Total pore volume

^d^Micropore volume

^e^Mesopore volume

### Electrochemical Performance

**Fig. 3:**
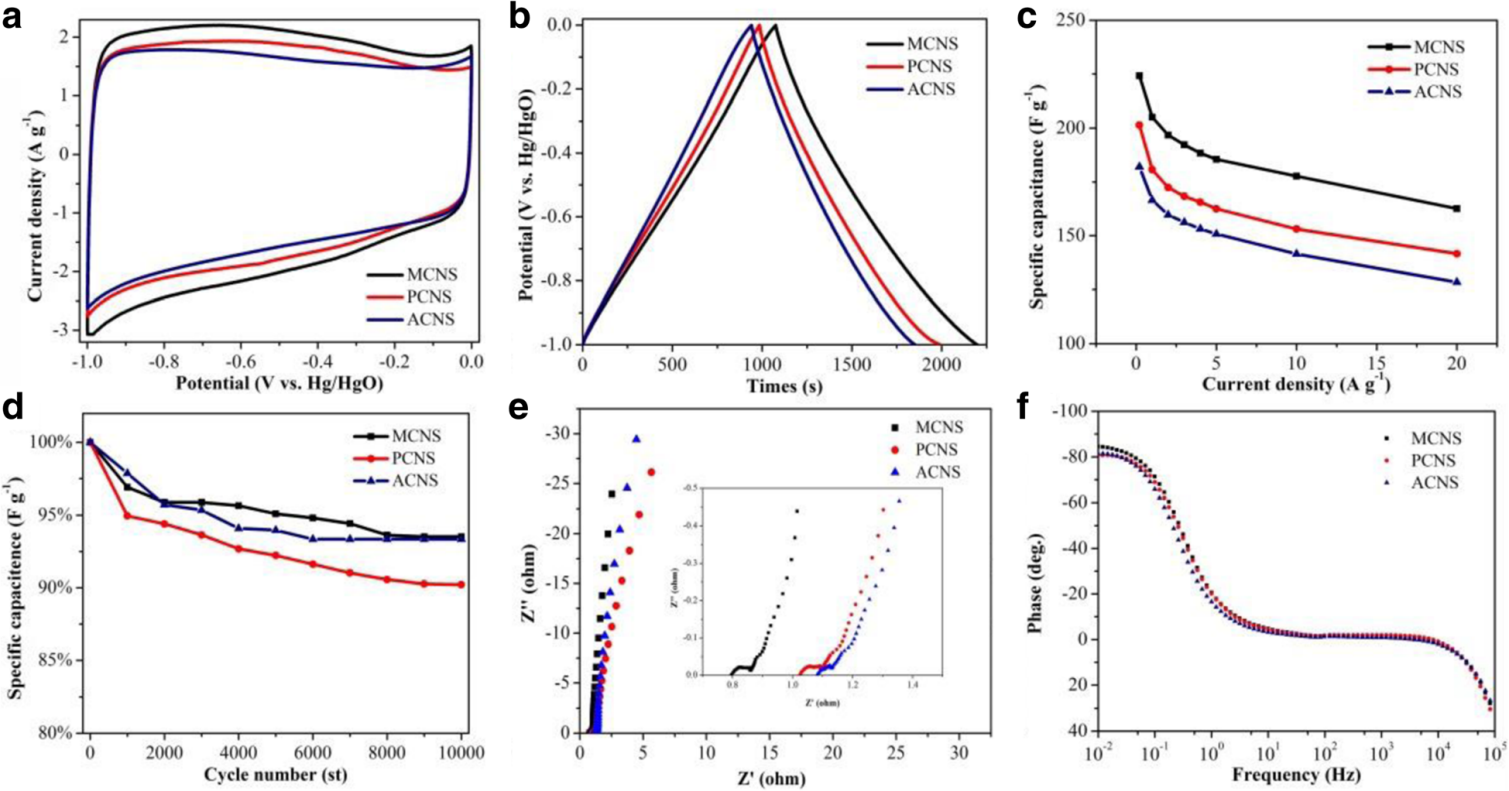
Electrochemical performance of MCNS, PCNS, and ACNS. **a** CV curves at 10 mV s^−1^. **b** CP curves at 0.2 A g^−1^. **c** Specific capacitance at different current densities. **d** Cycling test at 10 A g^−1^. **e** Nyquist plots in the frequency range from 10 mHz to 10 kHz. **f** Bode angle plots

As shown in Fig. 3, the electrochemical performance of MCNS, PCNS, and ACNS were evaluated and compared. The typical CV curves of different samples at 10 mV s^−1^ are shown in Fig. 3a. The quasi-rectangle shape with some broadened hump of CV curves is the synergetic effect of the electric double layer capacitance and pseudo-capacitance [17]. The bigger surrounding area of CV curve of MCNS indicates that the specific capacitance of MCNS is higher than that of PCNS and ACNS. Figure 3b compares the CP curves of different samples at 0.2 A g^−1^. The calculated specific capacitance of MCNS (224 F g^−1^) is larger than that of PCNS (201 F g^−1^) and ACNS (182 F g^−1^). The specific capacitance was calculated by CP curves at different current densities (Fig. 3c). At 20 A g^−1^, MCNS, PCNS, and ACNS show 72.7, 70.6, and 70.5% retention of the specific capacitance. The higher specific capacitance and better rate capability of MCNS can be attributed to superior structure of MCNS than of PCNS and ACNS. The mono-dispersion spheres create significant mesopore which could enlarge the electrode/electrolyte interface for transfer reaction and also serve as “ion buffering reservoir” for high-rate delivery. Also, the slightly mesopores inside carbon spheres are critical to afford less limited diffusion pathway for mass transport. Furthermore, the developed micropores provide large surface area to electrolyte ion for effective charge accumulation. Moreover, the agglomerated carbon spheres (ACNS) exhibit the hierarchical porous structure and enlarged specific surface area. Compared to MCNS, the electrochemical performance of ACNS is reduced. The result shows the importance of mono-dispersion spheres on enhancing the electrochemical performance. Obviously, the synergetic effect between mono-dispersion spheres and hierarchical porous structures contribute to the better electrochemical performance of MCNS. Figure 3d presents the results of cycling test at 10 A g^−1^ for 10,000 cycles. Over the 10,000 cycles, 93, 90, and 93% of the initial capacitance were retained for MCNS, PCNS, and ACNS, respectively. The Nyquist plot was given by EIS tests, as shown in Fig. 3e. The equivalent series resistance (ESR) values of MCNS (0.76 Ω) is smaller than that of PCNS (1.02 Ω) and ACNS (1.08 Ω), indicating the better electrical conductivity of MCNS. Moreover, from Fig. 3f, the phase angle of MCNS, PCNS, and ACNS are close to − 90° for ideal capacitor [18]. In detail, the phase angle of MCNS, PCNS, and ACNS are − 84.5°, − 80.5°, and − 81.4°, respectively. In overall consideration of electrochemical performance, the MCNS are better than the PCNS and ACNS. Thus, such MCNS shows great potential as electrode material for supercapacitors.

## Conclusions

With increasing dosage of F108, three different carbon spheres, polydisperse carbon nanospheres (PCNS), monodisperse carbon nanospheres (MCNS), and agglomerated carbon spheres (ACNS), were successfully obtained. The porous structure difference between three carbon spheres is mainly caused by the adding of F108. The prepared MCNS are uniform particle size with hierarchical pore structure while the PCNS show a wide size distribution and microporous structure, but the ACNS are firmly aggregated and nondispersible. MCNS, PCNS, and ACNS exhibited different electrochemical performance. The synergetic effect of mono-dispersion spheres and hierarchical porous structures contributes to the better electrochemical performance of MCNS. Compared to PCNS and ACNS, the as-prepared MCNS show the highest specific capacitance of 224 F g^−1^ at 0.2 A g^−1^, the best rate capability, and the most excellent capacitance retention of 93% over 10,000 cycles, which makes it to be the candidate for high-performance supercapacitors.

## Abbreviations

ACNS: Agglomerated carbon nanospheres
CP: Chronopotentiometry
CV: Cyclic voltammetry
EIS: Electrochemical impedance spectroscopy
ESR: Equivalent series resistance
mCNS: Intermediate carbonized carbon nanospheres for MCNS
MCNS: Monodisperse carbon nanospheres
PCNS: Polydisperse carbon nanospheres
